# Reduced postprandial energy expenditure and increased exogenous fat oxidation in young woman after ingestion of test meals with a low protein content

**DOI:** 10.1186/1743-7075-5-25

**Published:** 2008-10-17

**Authors:** Klaus J Petzke, Susanne Klaus

**Affiliations:** 1 Group of Stable Isotopes and of Energy Metabolism, German Institute of Human Nutrition in Potsdam-Rehbruecke (DIfE), Arthur-Scheunert-Allee 114-116, 14558 Nuthetal, Germany

## Abstract

**Background:**

Macronutrient composition of diets can influence energy balance in humans. We tested the hypothesis whether low protein content in single meals may induce lower values of energy expenditure (EE) and fat oxidation (FO) as compared to adequate protein content.

**Methods:**

Indirect calorimetry was combined with a breath test using naturally ^13^C-enriched corn oil to differentiate between postprandial exogenous and endogenous FO. Young women ingested single meals containing either 3.9% (low protein, LP) or 11.4% (adequate protein, AP) of total energy (~3100 kJ) as protein.

**Results:**

Postprandial EE was 160 kJ/6 h lower (p < 0.01) after LP meals and diet induced thermogenesis (DIT) increased less (p < 0.001) as compared to AP meals. Total postprandial FO was not significantly different between meals (~17 g/6 h). However, exogenous postprandial FO was significantly (p < 0.01) higher (4.28 ± 1.57 g/6 h) after exposure to LP meals as compared to AP meals (1.87 ± 1.00 g/6 h). Less than 10% of ingested fat (50 g) was oxidized in the postprandial phase. The overall postprandial fat balance was approximately + 33 g.

**Conclusion:**

Breath tests using naturally ^13^C-labeled corn oil mirror exogenous FO. Low protein meals resulted in reduced postprandial EE and increased exogenous FO as compared to adequate protein meals without differences in total FO.

## Background

Obesity is an increasing health problem in both developed and developing countries [1]. Genetic predisposition, physical inactivity, and the consumption of high-energy dense foods are risk factors for obesity [2]. It was shown that diet composition influences energy storage in the body which supported the view that macronutrients may play a role in the development of obesity. However, the results are contradictory and it remains unclear what the relative importance and metabolic advantages of protein, carbohydrate and fat are [3,4]. Therefore, studies of in vivo effects of macronutrients on body fat storage and metabolism could enhance our understanding of the aetiology of obesity and improve methods for body-weight management.

In order to achieve a reduction in body fat, weight reducing diets must attain a negative energy or fat balance. An increase of the protein content of diets at the expense of carbohydrates has brought such an advantage [4-6]. Fat oxidation (FO) was shown to depend on dietary composition, being higher after a relatively high protein meal as compared to a balanced diet [7]. Furthermore, there is increasing evidence of higher energy expenditure (EE) following protein ingestion as compared to carbohydrates or fat [8-12]. However, the mechanisms for the influence of dietary protein on energy expenditure are not clear and controversially discussed [6,13,14].

The combination of indirect calorimetry with isotope techniques is an effective tool for the examination of the oxidation of endogenous and ingested nutrients in vivo [15-19]. As demonstrated in recent reports ^13^CO_2 _breath tests are now widely used in metabolic studies of nutrients using stable isotopic labeled precursors [20,21]. 10 to 30% of ingested fat were reported to be oxidized postprandially in subjects exposed to different experimental conditions usually using fat supplemented with ^13^C-labeled fatty acids [15-17]. Only few studies were published using ^13^C-labeled triglycerides as tracers [22]. However, there are no reports in the recent literature available using non supplemented naturally enriched fat such as corn oil to study FO. The use of uniformly labeled naturally enriched fat in metabolic studies may have the advantage of avoiding artefacts due to possible differences in metabolism of artificially with stable isotopes labeled precursors as it was shown e. g. for crystalline amino acids compared to protein-bound amino acids [23]. Therefore, we examined in a pilot study the effects of different test meals with low or adequate protein content and an equal fat content on postprandial EE and substrate oxidation. We hypothesized that an exposure of subjects to test meals with lower protein content may cause reduced rates of EE and FO with possible consequences on energy balance. Exogenous FO was measured using corn oil naturally enriched in ^13^C-content as the only fat component.

## Methods

### Subjects

6 healthy young women volunteered for the study (Table 1). They were students from Potsdam University and took part in a traineeship of studies in physiology of nutrition. Participants reported to be non smokers, normo-glycemic, and normo-lipidemic, and free of any endocrine or organ disorders. Furthermore, women were neither pregnant nor lactating and between 3 and 10 d after menstruation. The body mass index (BMI, in kg/m^2^) was within normal range for all of the subjects and they did not perform any extreme physical activities. Baseline breath ^13^CO_2 _enrichments were characteristic for a C_3 _plant based habitual diet [24]. All subjects volunteered gave an informed consent before participation in the study.

**Table 1:** General characteristics of the study participants

	Mean (n = 6) ± SD
Age (y)	25.5 ± 2.6
Weight (kg)	59.8 ± 8.3
Height (m)	1.7 ± 0.1
BMI (kg/m^2^)	20.6 ± 2.5
Baseline δ^13^C in breath CO_2 _[‰ vs. PDB]	-25.8 ± 0.7

### Test meals

Composition and sources of the test meals are provided in Table 2 and Table 3. The meals were comparable in energy content but either low (LP, 3.9% of energy) or adequate (AP, 11.4% of energy) in protein content. The terms "low" or "adequate" is based on the Recommended Dietary Allowance for men and women of 0.8 g good quality protein/kg body weight/day. This amount of protein corresponds to about 10% of the energy content of a balanced mixed diet [25]. The carbohydrate content was either 35.4 or 27.8% of energy in the LP or AP test meal, respectively. The total fat content was about 60% of energy in both test meals and consisted practically exclusively of C_4 _plant based corn oil. ^13^C abundance of test meal components was determined (Table 2). The low ^13^C abundance (expressed as δ^13^C vs. PeeDee Belemnite Limestone carbonate, PDB), see below) of skimmed milk powder showed that animals were not fed with corn based food. Furthermore, the ^13^C abundance of saccharose is characteristic for beet sugar. Both of these components are C_3 _plant-based. This is an important condition to measure ingested FO using C_4 _plant-based naturally ^13^C-enriched corn oil. As shown previously [26] and tested prior to the beginning of this study, C_3 _based low ^13^C meal components have no effect on baseline breath ^13^CO_2 _production [Unpublished observations by Petzke]. ^13^C abundance of corn oil was by about 10 to 12 ‰ δ^13^C higher than that of the other macronutrient components of the test meals (Table 2). All solid components of each meal were of similar weight (130 g). All components were mixed with water free of carbonic acid to reach a mushy consistency and an equal volume (250 mL). Meals were ingested after baseline measurement of energy expenditure (EE) within 10 min at the beginning of the study at 09:00 to 09:30 h. Additional drinking of mineral water free of carbonic acid was allowed during the study protocol.

**Table 2:** Composition and sources of test meals

Test meal		LP	AP	δ^13^C [‰ vs. PDB]^5^
Skimmed milk powder^1^	(g)	20	60	-28.2 ± 0.7
	(kJ)	297	895	
Corn oil^2^	(g)	50	50	-16.1 ± 0.2
	(kJ)	1881	1881	
Wheat starch^3^	(g)	40	0	-26.9 ± 0.2
	(kJ)	581	0	
Saccharose^4^	(g)	20	20	-27.8 ± 0.2
	(kJ)	334	334	

^1^1492 kJ/100 g, Naturaflor^®^, Töpfer GmbH, Dietmannsried, Germany

^2^3762 kJ/100 g, Mazola^®^, Unilever Deutschland, Hamburg, Germany

^3^1450 kJ/100 g, Weizella, Hermann Kröner Weizenstärkefabrik, Ibbenbüren, Germany

^4^1668 kJ/100 g, Zucker, Nordzucker, Braunschweig, Germany

^5^Data are mean values ± SD, n = 5 measurements.

**Table 3:** Macronutrient composition of test meals^1^

Test meal	LP	AP
Energy content (kJ)	3114	3131
		
Protein (g)	7.2	21.0
Protein (kJ)	121	357
Protein (% total energy)	3.9	11.4
		
Total fat (g)	50.2	50.6
Total fat (kJ)	1890	1903
Total fat (% total energy)	60.7	60.8
		
Carbohydrate (g)	64.9	51.6
Carbohydrate (kJ)	1102	872
Carbohydrate (% total energy)	35.4	27.8

^1^Based on calculations using [Food Composition and Nutrition Tables, 5^th ^revised and completed version, compiles by Schertz H and Senser F, medpharm Scientific Publishers, Stuttgart, 1994]

### Study protocol and calorimetry

Studies were performed after an overnight (12 h) fast. All participants took part twice and consumed both test meals randomly in a distance of 2 days in between. All subjects underwent measurements of EE using an open-circuit indirect calorimeter with a ventilated-hood system (Deltatrac™ II, Datex Instrumentarium Corp., Helsinki, Finland). The system was calibrated daily according to the instructions using 5% CO_2 _in O_2 _calibration gas (QUICK CAL™, GE Healthcare Finland Oy, Helsinki). After a run-in period of 30 min, energy expenditure (EE) was determined between 08:00 and 09:00 within 30 min. Then, subjects ingested the test meals over a period of 10 min and postprandial EE was measured three times after 30, 150, and 270 min of the study protocol. Breath samples were collected 5-fold at baseline (-10 min) and twice every 30 min for a period of 6 h after the meal into tubes (10 mL Exetainer^®^, Labco Ltd., Buckinghamshire, U.K.) for measurement of breath^13^CO_2 _enrichments. The bladder was emptied before the study. During the experimental period within a total time interval of 360 min, complete urine samples were collected for the assessment of nitrogen excretion. Nitrogen content in urine was determined by Kjeldahl method (Kjeldatherm-Turbosog-Vapodest 45, C. Gerhardt GmbH & Co. KG, Bonn, Germany). For each measurement of EE the first 5 to 10 min were discarded to allow subjects to adapt to the measurement procedure, and data from the remaining 20 min were averaged and used to calculate EE and substrate oxidation based on oxygen consumption, carbon dioxide production, and urinary nitrogen excretion.

### Stable isotopic analysis

The bulk ^13^C abundances in food components were determined using an elemental analyser (EA 1108, Fisons Instruments, Rodano, Italy) coupled on-line via a conflo interface to an isotope-ratio mass spectrometer (EA/IRMS; Delta C, Thermo Electron GmbH, Bremen, Germany) as described [27]. Breath ^13^CO_2 _enrichments were analysed by isotope ratio mass spectrometry (BreathMAT, Thermo Electron GmbH). Isotopic compositions of carbon are reported in the conventional delta per mill notation in the range of natural abundance expressed in parts per thousand relative to the international standard PDB (δ^13^C in ‰ vs. PDB) as described [27]:

δ^13^C (‰) = [R_sample_/R_standard _- 1] × 10^3^

where R_standard _= [^13^C]/[^12^C] = 0.0112372 for PDB that has been assigned a δ^13^C value of 0.0‰. R_sample _is derived from the ratios of the mass spectrometer ion currents ranging from *m/z *44 to *m/z *46.

### Calculations and statistics

EE (kcal per day) was calculated as

EE = 5.50 × VO_2 _+ 1.76 × VCO_2 _- 1.99 × *N*_ex_

where VO_2 _is the oxygen consumption (Liters per day), VCO_2 _is the CO_2 _production (Liters per day) and N_ex _is the urinary nitrogen excretion (grams per day) [28]. Respiratory quotient (RQ) is defined as VCO_2 _divided by VO_2_. DIT was calculated by obtaining the increment in postprandial EE above pre-meal baseline values (Table 4) during the measurement period of the protocol as described previously [29]. DIT was expressed in absolute terms (kJ/6 h) and as a percentage of the energy in the test meals. Whole body substrate oxidation rates were calculated before (fasting) and during 6 h after test meal ingestion (postprandial) based on measurements of EE at 30, 150, and 270 min of the study protocol. Net rates of FO, carbohydrate oxidation (CO), and protein oxidation (PO) (in grams per day) were calculated as [30]:

**Table 4:** Baseline measurements before ingestion of test meals (n = 6)

Test meal	LP	AP
EE (kJ/h)	218 ± 12	230 ± 13
RQ	0.87 ± 0.04	0.86 ± 0.08
		
PO (g/h)	2.73 ± 0.66	2.73 ± 0.66
FO (g/h)	2.72 ± 1.53	3.05 ± 2.53
CHO (g/h)	10.7 ± 2.9	8.65 ± 4.41

FO = 2.432 × VO_2 _- 2.432 × VCO_2 _- 1.943 × *N*_ex_

CO = 5.926 × VCO_2 _- 4.189 × VO_2 _- 2.539 × *N*_ex_

PO = 6.25 × *N*_ex_.

Ingested FO was estimated as reported by Sonko et al. [15,16] using the following equation:

Q_exFO _= [(δ_t _- δ_b_) + (δ_t-1 _- δ_b_)] × (Q_CO2 _× MW)/2(δ_s _- δ_b_) × 0.56 × n

where Q_exFO _is the quantity of ingested fat oxidized, δ_b _is the baseline breath ^13^CO_2 _abundance (‰ δ^13^C vs. PDB) δ_t _is the breath ^13^CO_2 _abundance at time *t *post meal ingestion, δ_t-1 _is breath ^13^CO_2 _abundance at any other time after *t*, δ_s _is the ^13^C abundance of labeled fat ingested, Q_CO2 _is the quantity of CO_2 _expired in breath (moles) for the time interval in question, *MW *is the average molecular weight (866 g/mol) of ingested corn oil triglycerides, and *n *(= 55) is the number of carbon atoms in corn oil triglyceride molecule. 0.56 is the acetate correction factor to account for label fixation that might occur at any step between the entrance of labeled acetyl-CoA into the tricarboxylic acid cycle until the recovery of label in breath CO_2 _[31].

Data are reported as means ± SD. Comparison of means was performed with two-sided paired t-test. Pearson correlation coefficients were calculated to determine the relationship between selected parameters. Differences with p < 0.05 were considered statistically significant. WinSTAT^® ^(vers. 1999.2, R. Fitch software, Staufen, Germany) was used to compute statistical analysis.

## Results

EE and DIT expressed in absolute terms (kJ/6 h) or as percentage of energy in test meals were significantly lower after ingestion of LP meals compared to AP meals (Table 5). There was no change in RQ following both test meals and no significant difference in RQ between different test meals. The relatively high baseline RQ (Table 4) can not be satisfactorily explained. It could be that the overnight fasting period was not sufficient to deplete glycogen stores. Alternatively, the resting period of 30 min before measurement of the resting EE might not have been sufficient for young, healthy normal weight female subjects. As expected, a lower protein intake with the LP meal reduced postprandial PO in relation to fasting levels. On the other hand postprandial PO increased relative to fasting levels following AP meals. The change in PO between test meals was significantly different. Total postprandial CO was not significantly different (p > 0.05) after exposure to different test meals. Total postprandial FO was not significantly different between both test meals. However, postprandial FO slightly increased or decreased as compared to measurements during fasting after exposure to LP or AP meals, respectively. Interestingly, exogenous postprandial FO was significantly higher (p = 0.005) by about 2.4 g/6 h after ingestion of LP meals compared to AP meals. However, the total amount of oxidized exogenous fat during 6 h was relatively low (less than 10% compared to ingested fat mass). On the other hand, endogenous postprandial FO was not significantly different between young woman ingesting LP or AP test meals due to high individual variations. The overall postprandial fat balance was approximately + 33 g. Interestingly, DIT positively correlated with PO (R^2 ^= 0.739, p = 0.011), and exogenous FO negatively correlated with change in PO (R^2 ^= 0.532, p = 0.037).

**Table 5:** The influence of the test meals on postprandial energy expenditure and substrate oxidation (n = 6)

Test meal	LP	AP	p <
Postprandial EE (kJ/6 h)	1356 ± 69	1516 ± 66	0.01
DIT (kJ/6 h)^1^	46.5 ± 5.7	134.6 ± 34.3	0.001
DIT (%)	9.0 ± 1.1	25.9 ± 6.6	0.001
RQ	0.87 ± 0.04	0.87 ± 0.03	ns^4^
Change in RQ	-0.01 ± 0.06	0.01 ± 0.05	ns
Postprandial PO (g/6 h)	14.9 ± 4.20	18.0 ± 4.2	ns
Change in PO (g/6 h)^1^	-1.50 ± 1.23	1.55 ± 1.29	0.001
Postprandial CO (g/6 h)	55.6 ± 17.8	58.7 ± 14.1	ns
Change in CO (g/6 h)^1^	-8.70 ± 27.65	6.77 ± 25.34	ns
Postprandial FO (g/6 h)	16.9 ± 7.5	16.8 ± 8.8	ns
Change in FO (g/6 h)^1^	0.55 ± 7.91	-1.50 ± 7.00	ns
Endogenous postprandial FO (g/6 h)^2^	12.6 ± 7.8	14.9 ± 9.2	ns
Exogenous postprandial FO (g/6 h)^3^	4.28 ± 1.57	1.87 ± 1.00	0.01
Contribution of exogenous postprandial FO of energy expenditure (kJ/6 h; % of postprandial EE)	169; 12	74; 5	

^1^Calculated as sum of postprandial values over 6 h – (fasting value per h × 6).

^2^Calculated as exogenous FO – postprandial FO.

^3^Based on ^13^CO_2 _breath test.

^4^ns = p > 0.05.

**Figure 1:**
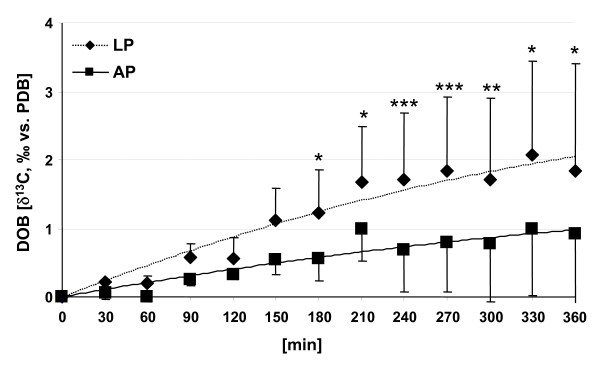
**Delta value over baseline (DOB) of breath ^13^CO_2 _of young woman after ingestion of test meals with either 3.9% (LP) or 11.4% (AP) of energy as protein and with naturally enriched corn oil as ^13^C source.** Data are means ± SD, n = 6. Values with indications are significantly different (*p < 0.05, **p < 0.01, ***p < 0.001). For meal composition and details, see method section.

Baseline breath δ^13^CO_2 _values (Table 1) were similar to those of C_3 _plant based nutrients (Table 2, [22]). In response to test meal ingestion it was evident that DOB values of breath ^13^CO_2 _progressively increased in all subjects (Figure 1). A peak value of ^13^C enrichment in breath ^13^CO_2 _was not reached during 6 h of the study protocol. DOB values increased within 6 h up to 1 or 2 ‰ δ^13^C after ingestion of AP- or LP-meals, respectively. The δ^13^C values in breath CO_2 _of young woman were significantly different (p < 0.05) from 180 min and thereafter comparing between the exposure to LP and AP meals.

**Figure 2:**
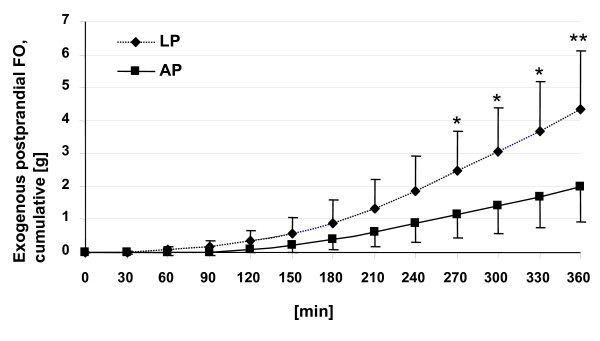
**Cumulative exogenous fat oxidation (FO) of young woman after ingestion of test meals with either 3.9% (LP) or 11.4% (AP) of energy as protein and with naturally enriched corn oil as ^13^C source.** Data are means ± SD, n = 6. Values with indications are significantly different (*p < 0.05, **p < 0.01). For meal composition and details, see method section.

The time course of cumulative exogenous postprandial FO calculated from δ^13^CO_2 _breath values is shown in Figure 2. Cumulative exogenous postprandial FO based on ^13^CO_2 _production from corn oil was significantly higher (p < 0.05) as of 270 min and thereafter of young woman exposed to the LP meal as compared to AP meal.

## Discussion

In the present pilot study we examined the effect of single ingestions of different test meals with either low or higher but adequate protein content on postprandial energy metabolism and FO in young woman. This was performed to test our hypothesis that an exposure to low protein containing meals may lead to reduced rates of EE and FO as compared to adequate protein meals which may have consequences on energy balance. Our results confirmed that dietary protein may influence postprandial EE and DIT. The postprandial EE was significantly higher in woman after exposure to the AP meal (protein content of 11.4% of energy) as compared to the LP meal (protein content of 3.9% of energy). The value of postprandial EE of young woman was approximately by 160 kJ/6 h higher after exposure to the AP meal as compared to the LP meal. This accounts to approximately 5% of the energy content of test meals. Therefore, the present results confirmed former studies which have shown that protein can induce a higher thermic effect compared to other macronutrients and that high protein diets have a higher potential to increase EE than low protein diets [10,32,33]. However, data in literature are not uniform. It was shown under conditions of energy-restriction that a short-term replacement of dietary carbohydrate with protein did not increase the thermic effect of food to facilitate weight loss in type 2 diabetes patients [34]. Moreover, overfeeding of either low- or high-protein diets were found to increase DIT as compared to AP diets [12]. Although, habitual energy intake of young woman was not evaluated in our present pilot study, we assumed no restriction or excess in food energy consumption before test meal consumption. In future studies of postprandial effects of protein consumption an evaluation of the role of previous energy intake will be necessary because food energy deficiency was supposed to prevent thermic effects of dietary proteins [35]. The mechanisms regulating thermic effects after consumption of high protein diets remain to be elucidated. However, based on the prevention of thermic effects in energy deficient states a role of mitochondrial metabolism in modifying energy efficiency may be supposed [35].

In addition, it has been shown that subjects consuming high protein diets (30% of energy) for 4 d exhibited an increased EE and higher rates of FO resulting in lower body fat mass when fed at energy balance and compared to diets containing an adequate protein content (10% of energy) [33,36]. In the present study we did not find differences in total FO in subjects exposed to LP or AP test meals. Interestingly, exogenous postprandial FO was significantly lower in subjects exposed to AP meals compared to LP meals. It was shown in former studies that net lipid oxidation is dependent on short-term changes in dietary intake, being significantly higher after a high protein meal compared to a meal relatively balanced in macronutrient composition [7]. The reason for lower rates of exogenous FO after exposure to AP meals compared to LP meals is not clear. However, lower rates of gastric emptying after administration of diets containing higher proportions of milk proteins can not be excluded and have to be evaluated in future studies [37]. Taken together, it seems that although able to exhibit higher rates of exogenous FO, meals with a relatively low protein content are not advantageous compared to adequate protein meals with respect of lowering total energy or fat balance.

The measurement of enrichment in expired ^13^CO_2 _is widely used to determine nutrient oxidation rates using different infusion techniques or oral consumption of ^13^C-labeled substrates. Mostly substrates are applied which were exogenously labeled with ^13^C as fatty acids to enrich the precursor pools by supplementation to fat of test meals to increase the outcome of ^13^CO_2 _enrichment in breath samples. However, fatty acids may be metabolized differently than nutrients like fat usually consumed with complex diets. Therefore, intrinsically or naturally enriched tracers should be preferred. Analytical techniques are now sensitive enough to detect relatively small changes of enrichments in the range of natural abundances. Corn oil is naturally enriched (about -16 ‰ δ^13^C) due to discriminatory accumulation of ^13^C during photosynthesis. Naturally ^13^C-labeled triglycerides from corn oil are absorbed and cleaved by lipolysis to ^13^C-labeled fatty acids and glycerol before incorporation into endogenous fat deposits or subsequent mitochondrial β-oxidation, which results in the production of ^13^CO_2 _in breath. Therefore, naturally ^13^C-enriched corn oil can be used to examine exogenous FO when the subject's habitual diet and test meal components do not contain other corn based products. In the present experiment we have shown that a supplementation of corn oil with ^13^C-fatty acids was not required. This avoids possible artefacts due to different absorption and metabolism of fatty acids as compared to triglycerides normally available from dietary fat. In addition, no additional assumptions are necessary with respect to not uniformly labeled fatty acids usually supplemented as a tracer. The calculation of exogenous postprandial FO was possible although the mean increase of δ^13^C values over baseline in breath CO_2 _amounted to only 1 to 2 ‰ vs. PDB. The method permitted a significant differentiation of effects due to differences in test meal composition on FO. However, it was an essential preposition that baseline values of ^13^CO_2 _enrichment did not change during experimental period which we have tested using sunflower seed oil instead of corn oil [Unpublished observations by Petzke].

## Conclusion

We have shown that corn oil naturally enriched with ^13^C can be used to evaluate postprandial FO in vivo. Furthermore, we provide evidence that the amount of dietary protein in relation to other macronutrients may influence postprandial exogenous and endogenous FO the consequences and mechanisms of action of which has to be examined in future studies.

## Abbreviations

AP: Adequate protein; BMI: Body mass index; CHO: Carbohydrate oxidation; DIT: Diet induced thermogenesis; DOB: Delta value over baseline; EE: Energy expenditure; EA/IRMS: Elemental analyser/isotope ratio mass spectrometer; FO: Fat oxidation; LP: Low protein; PDB: PeeDee Belemnite Limestone carbonate; PO: Protein oxidation; RQ: Respiratory quotient.

## Competing interests

The authors declare that they have no competing interests.

## Authors' contributions

KJP has contributed to the design of the experiment and conducted and directed the sample and data analysis. Furthermore, he was involved in the writing and editing of this manuscript. SK directed the research and contributed to the preparation of the manuscript.
